# Validation of Laboratory Risk Indicator for Necrotizing Fasciitis (LRINEC) score and establishment of novel score in Japanese patients with necrotizing fasciitis (J‐LRINEC score)

**DOI:** 10.1111/1346-8138.17663

**Published:** 2025-02-07

**Authors:** Yuta Norimatsu, Takemichi Fukasawa, Yuki Ohno, Yurie Norimatsu, Kazuki M. Matsuda, Teruyoshi Hisamoto, Hirohito Kotani, Ai Kuzumi, Asako Yoshizaki‐Ogawa, Takuya Miyagawa, Koji Oba, Shinichi Sato, Ayumi Yoshizaki

**Affiliations:** ^1^ Department of Dermatology JR Tokyo General Hospital Japan; ^2^ Department of Dermatology The University of Tokyo Graduate School of Medicine Tokyo Japan; ^3^ Department of Dermatology International University of Health and Welfare Narita Hospital Chiba Japan; ^4^ Department of Clinical Cannabinoid Research The University of Tokyo Graduate School of Medicine Tokyo Japan; ^5^ Department of Biostatistics, School of Public Health, Graduate School of Medicine The University of Tokyo Tokyo Japan

**Keywords:** cellulitis, Laboratory Risk Indicator for Necrotizing Fasciitis (LRINEC) score, Laboratory Risk Indicator for Necrotizing Fasciitis for Japanese Patients [J‐LRINEC], necrotizing fasciitis, phlegmon

## Abstract

The Laboratory Risk Indicator for Necrotizing Fasciitis (LRINEC) score is widely used to distinguish between necrotizing fasciitis and cellulitis. However, LRINEC scores are not as sensitive or specific as initially reported, possibly due to differences in patient backgrounds in different countries. Here, we examined the validity of LRINEC scores in Japanese patients. We also investigated the possibility of developing a new scoring system. Patients with necrotizing fasciitis (*n* = 56) and cellulitis (*n* = 209) were retrospectively evaluated. The data were split into training (*n* = 199) and validation (*n* = 66) datasets. A logistic regression analysis was used to calculate the C‐statistics of the LRINEC scores. A new equation was formulated using logistic regression analysis with an appropriate variable selection (Laboratory Risk Indicator for Necrotizing Fasciitis for Japanese Patients [J‐LRINEC] score). The J‐LRINEC score had a C‐statistic of 0.9683, sensitivity of 91.4%, and specificity of 84.8%. The LRINEC score had a C‐statistic of 0.914 and specificity of 96%; however, its usefulness was limited by its sensitivity of 68.9%. Our results suggest that the LRINEC score is valid for Japanese patients; however, the J‐LRINEC score showed higher sensitivity and specificity, suggesting that it may be a useful tool for differentiating cellulitis from necrotizing fasciitis among Japanese patients.

## 1 INTRODUCTION

Cellulitis, a recurrent infectious skin disease is commonly encountered by dermatologists in clinical practice, is characterized by edema, a burning sensation, and pain.^1^ The differential diagnosis of cellulitis is broad^2^ and includes both non‐infectious conditions, such as stasis dermatitis and contact dermatitis, and infectious conditions, such as necrotizing fasciitis, an important differential diagnosis of cellulitis. Necrotizing fasciitis is an infectious skin disease characterized by erythema, edema, and pain; however, unlike cellulitis, it spreads rapidly and has a high mortality rate.^3^ Additionally, making a skin incision, an important step in the treatment of necrotizing fasciitis, is an invasive procedure.^2^ Therefore, it is important to differentiate between the two conditions.

Necrotizing fasciitis is common in patients with diabetes, liver failure, peripheral arterial disease, immunodeficiency, and a surgical history.^3^, ^4^ In contrast, the risk factors for cellulitis include a history of cellulitis, toe web infection with *Staphylococcus aureus* or beta‐hemolytic streptococcus, ringworm infection, obesity, lymphedema, peripheral circulatory disturbance, history of surgery, psoriasis, and scars. Therefore, it is often difficult to distinguish between these two conditions based on a patient's medical history alone.^5^, ^6^, ^7^, ^8^, ^9^

The Laboratory Risk Indicator for Necrotizing Fasciitis (LRINEC) score is widely used to differentiate cellulitis from necrotizing fasciitis. It has very high positive (92%) and negative (96%) predictive values. The LRINEC score is not only useful for early diagnosis, but has also been used to determine the severity of necrotizing fasciitis since it has been suggested that patients with an LRINEC score of ≥6 have a longer hospital stay than those with an LRINEC score of <6.^10^ An article published in *The New England Journal of Medicine* (NEJM) in 2017 also used the LRINEC score for diagnosis.^11^ The LRINEC score is also an important tool for diagnosing necrotizing fasciitis.^11^, ^12^, ^13^ In addition to the LRINEC score, the NEJM algorithm was used to detect necrotizing fasciitis in patients with tachycardia (heart rate >120 bpm), hypotension, an elevated creatine kinase level, and a C‐reactive protein (CRP) level of >15 mg/dL. However, the LRINEC score may not be as sensitive and specific as initially reported,^14^, ^15^, ^16^, ^17^ possibly because of differences in patient characteristics in different countries.^14^, ^15^, ^16^, ^17^

In recent years, new scores for differentiating cellulitis from necrotizing fasciitis, such as the Site other than lower limb, Immunosuppression, Age ≤60 years, Renal impairment, and Inflammatory markers (SIARI) score, have been developed in various countries.^18^, ^19^ The characteristics of cellulitis reportedly differ between patients in Japan and patients in other countries.^20^, ^21^ Therefore, the LRINEC score may not be as accurate as originally reported for Japanese patients, for whom a new score may be required.

In this study, we examined the possibility of developing a tool that can differentiate cellulitis from necrotizing fasciitis among Japanese patients.

## 2 MATERIALS AND METHODS

We retrospectively reviewed the data of patients with necrotizing fasciitis or cellulitis who were hospitalized and treated at the JR Tokyo General Hospital and University of Tokyo Hospital between April 1, 2005, and March 31, 2018. Necrotizing fasciitis and cellulitis were diagnosed based on clinical, imaging, and laboratory findings. Patients for whom data were missing or who had osteomyelitis or pressure ulcer infections were excluded.

Non‐normally distributed variables were analyzed using the Mann–Whitney *U* test, and categorical data were analyzed using Fisher's exact test or the chi‐squared test. All tests were two‐tailed and statistical significance was set at *p* = 0.05. Patients for whom many data points were missing, such as those for whom blood tests were not performed, were excluded from the analysis. Age, body mass index (BMI), sex, white blood cell (WBC) count, red blood cell count, platelet count, CRP, hemoglobin, sodium, and albumin levels, immunosuppression due to any cause (positive/negative), infection site (leg) (positive/negative), pain (positive/negative), temperature, pulse rate, signs of acute kidney injury (positive/negative), history of ulcer (positive/negative), and blisters (positive/negative) were analyzed. The data were randomly split into training (*n* = 199) and validation (*n* = 66) datasets. A logistic regression analysis was used to calculate the C‐statistics for the LRINEC, SIARI, and NEJM algorithms. A new equation was formulated using logistic regression analysis with the forward selection method. Specifically, we examined whether there was an interaction between age and other variables when creating the new equation we call the Laboratory Risk Indicator for Necrotizing Fasciitis for Japanese Patients (J‐LRINEC).

The ethics review boards of the University of Tokyo and JR Tokyo General Hospital (approval no. 695) approved the study design. This study complies with the principles of the Declaration of Helsinki. An opt‐out consent form was used. All patients were informed that their information could be used for future presentations or publications, either through the institutional criteria or a research consent form.

## 3 RESULTS

### 3.1 Patient characteristics

This study included 56 patients with necrotizing fasciitis and 209 with cellulitis. The patients' characteristics are presented in Table 1. No intergroup difference was observed in the sex ratio. However, those with necrotizing fasciitis tended to be older and have a lower BMI than those with cellulitis. Patients with necrotizing fasciitis were also significantly more likely to be immunosuppressed than those with cellulitis. The physical examination findings are shown in Table 2. Necrotizing fasciitis tended to occur in areas other than the lower extremities. The vital parameters tended to be worse in patients with necrotizing fasciitis versus cellulitis. Necrotizing fasciitis was more likely to cause blisters than cellulitis. The blood test results are shown in Table 3. Patients with necrotizing fasciitis had higher leukocyte counts and blood glucose, hemoglobin A1c, and CRP levels and lower hemoglobin, sodium, and protein levels and erythrocyte counts than those with cellulitis. Patients with necrotizing fasciitis were more likely to have renal dysfunction than those with cellulitis. Procalcitonin levels tended to be higher in patients with necrotizing fasciitis versus cellulitis, although the difference was not significant because of the small number of patients in whom these levels were measured.

**TABLE 1:** Characteristics of the patients.

	Necrotizing fasciitis (Mean ± SD)	Cellulitis (Mean ± SD)	*p*‐value
Age, years	61.82 ± 11.24	54.49 ± 19.17	0.0028
Body mass index, kg/m^2^	23.50 ± 5.337	25.95 ± 6.954	0.0132
Men: women	36:20	141:68	0.7495
Immunosuppression (+:–)	18:38	25:184	0.0008

*Note*: The analyses were performed using Fisher's exact test or the Mann–Whitney *U* test.

Abbreviations: −, negative; +, positive; SD, standard deviation.

**TABLE 2:** Physical examination findings of the patients.

	Necrotizing fasciitis	Cellulitis	*p‐* value
Lower extremity as the site of infection (+:–)	36:20	171:38	0.01
Pain (+:–)	46:2	158:35	0.0137
Body temperature (°C)	37.53 ± 1.016	36.99 ± 0.8985	0.0002
Heart rate (bpm)	94.02 ± 17.93	83.96 ± 16.03	0.0012
Skin barrier problems, ulcer (+:–)	41:14	125:81	0.0604
Blood pressure (mmHg)	120.6 ± 23.99	128.4 ± 17.02	0.0754
Blister (+:–)	18:36	24:177	0.0006

*Note*: Data are presented as mean ± SD except where otherwise indicated. The analyses were performed using Fisher's exact test or the Mann–Whitney *U* test.

Abbreviations: bpm, beats per minute; SD, standard deviation; −, negative; +, positive.

**TABLE 3:** Blood test results of the patients.

Parameter	Necrotizing fasciitis (mean ± SD)	Cellulitis (mean ± SD)	*p‐* value
White blood cells (/μL)	17 498 ± 8816	10 472 ± 4549	<0.0001
Hemoglobin (g/dL)	11.63 ± 1.937	13.45 ± 1.806	<0.0001
Red blood cells (×10^4^/μL)	383.5 ± 66.20	435.5 ± 66.49	<0.0001
Platelets (×10^4^/μL)	261.5 ± 146.7	248.0 ± 86.91	0.7354
Sodium (mEq/L)	134.9 ± 5.309	138.1 ± 3.318	<0.0001
Potassium (mEq/L)	4.133 ± 0.6083	4.045 ± 0.3885	0.5372
Chlorine (mEq/L)	98.73 ± 6.716	102.3 ± 3.749	<0.0001
Glucose (mg/dL)	224.2 ± 123.6	150.9 ± 88.73	0.0005
Hemoglobin A1c (NGSP) (%)	8.696 ± 2.878	6.777 ± 1.909	0.0031
Blood urea nitrogen (mg/dL)	28.55 ± 19.40	15.23 ± 10.23	<0.0001
Creatinine (mg/dL)	1.662 ± 1.710	0.8724 ± 0.5029	0.0026
Acute renal failure (+:–)	20:35	7:179	<0.0001
Total bilirubin (mg/dL)	0.8713 ± 0.6321	0.7943 ± 0.5211	0.4168
Aspartate transaminase (IU/L)	54.53 ± 131.2	30.13 ± 20.57	0.2737
Alanine transaminase (IU/L)	37.09 ± 58.14	29.45 ± 22.63	0.6463
Lactate dehydrogenase (IU/L)	307.5 ± 151.6	239.5 ± 68.71	0.0021
γ‐glutamyl transpeptidase (IU/L)	83.18 ± 127.7	55.89 ± 70.99	0.0482
Total protein (g/dL)	6.239 ± 0.8289	7.122 ± 0.6916	<0.0001
Albumin (g/dL)	2.798 ± 0.6971	3.809 ± 0.5580	<0.0001
Creatine kinase (IU/L)	284.4 ± 812.7	150.9 ± 234.0	0.9398
C‐reactive protein (mg/dL)	21.29 ± 10.94	7.865 ± 7.332	<0.0001
Procalcitonin (ng/ml)	40.81 ± 92.73	0.0900 ± 0.05657	0.0556
Anti‐streptolysin O antibody	183.9 ± 228.9	208.8 ± 250.4	0.5996
Fibrinogen (mg/dL)	571 ± 173.6	583.2 ± 174.5	0.6047

*Note*: The analyses were performed using Fisher's exact test or the Mann–Whitney U test.

Abbreviations: NGSP, National Glycohemoglobin Standardization Program; SD, standard deviation; −, negative; +, positive.

### 3.2 J‐LRINEC score

The newly developed equation was as follows:

$$J-\text{LRINEC score}=1/\left\{1+\exp\;\left(–S\right)\right\}.$$

where *S* = (−2.19466839641172) + −0.0326642793846153 * “age (years old)” + −0.111283611908592 * “body mass index (kg/m^2^)” + 0.102666886155143 * “CRP level (mg/dL)” + 0.446802301715261 * “temperature (°C)” + −3.64811896715886 * “albumin (g/dL)”.

### 3.3 Validity

**FIGURE 1:**
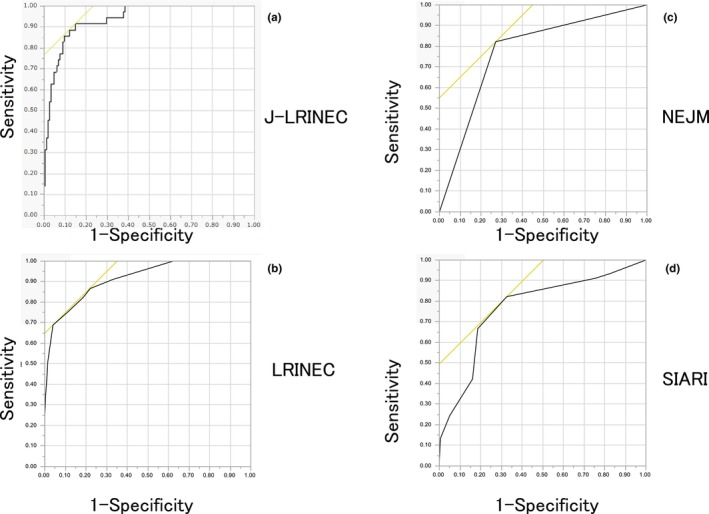
(a) The Laboratory Risk Indicator for Necrotizing Fasciitis for Japanese patients (J‐LRINEC) score has a C‐statistic of 0.9683, sensitivity of 91.4%, and specificity of 84.8%. The C‐statistic was 0.9683. (b) The LRINEC score has a C‐statistic of 0.914 and specificity of 96%, but its usefulness is limited by its sensitivity of 68.9%. (c) The New England Journal of Medicine (NEJM) algorithm has a C‐statistic of 0.775, sensitivity of 82.2%, and specificity of 72.9%. (d) The Site other than lower limb, Immunosuppression, Age ≤60 years, Renal impairment, and Inflammatory markers (SIARI) score has a C‐statistic of 0.769, sensitivity of 82.2%, and specificity of 67.4%.

For a J‐LRINEC score threshold of 0.1410 points, the sensitivity and specificity were 91.4% and 84.8%, respectively, while the C‐statistic was 0.9683. The receiver operating characteristic curves for the J‐LRINEC, LRINEC, NEJM, and SIARI scores are shown in Figure 1. Table 4 shows the sensitivity, specificity, and C‐statistics for each tool. The J‐LRINEC score showed the highest C‐statistic and the best sensitivity among the four tools, although its specificity was inferior to the LRINEC score (Figure 1, Table 4).

**TABLE 4:** Sensitivity, specificity, and C‐statistics for each tool.

	J‐LRINEC score	LRINEC score	NEJM algorithm	SIARI score
Sensitivity, %	91.4	68.9	82.2	82.2
Specificity, %	84.8	96	72.9	67.4
C‐statistic	0.9683	0.914	0.775	0.769

*Note*: NEJM algorithm: algorithm from Stevens and Bryant.^11^

Abbreviations: J‐LRINEC, Laboratory Risk Indicator for Necrotizing Fasciitis for Japanese patients; LRINEC, Laboratory Risk Indicator for Necrotizing Fasciitis; SIARI, Site other than lower limb, immunosuppression, age ≤60 years, renal impairment, and inflammatory markers.

## 4 DISCUSSION

The differences in the characteristics of Japanese patients with cellulitis versus necrotizing fasciitis, including LRINEC scores, WBC count, and CRP, hemoglobin, sodium, and creatinine levels, are shown in Tables 1, 2, 3. Hemoglobin, sodium, creatinine, and glucose levels, all of which are included in the LRINEC score, differed significantly between patients with necrotizing fasciitis and those with cellulitis. This result contrasts with that obtained for the modified LRINEC score, which does not include sodium level.^18^ This may reflect the differences in characteristics between European and Asian patients, as the modified LRINEC score was developed in Germany whereas the original LRINEC score was developed in Singapore.^13^, ^18^

Our SIARI score results showed that immunosuppression, infection site, renal function, WBC count, and CRP level were useful for differentiating necrotizing fasciitis from cellulitis among Japanese patients. According to a previous study that used the SIARI score, patients aged <60 years had a higher risk of necrotizing fasciitis than those aged >60 years.^19^ The logistic regression analysis also showed that the younger the age, the greater the risk of necrotizing fasciitis, suggesting that the SIARI score developed in New Zealand, which has the same temperate climate as Japan, is also valid and useful in Japanese patients.

In this Japanese study, the LRINEC score had a high C‐statistic of 0.914 and specificity of 96%, but its usefulness was limited by its sensitivity of 68.9%.^12^, ^13^ Therefore, in Japanese patients, the LRINEC score is useful for ruling out necrotizing fasciitis, but a positive result does not confirm its diagnosis. The NEJM algorithm had a C‐statistic of 0.775, sensitivity of 82.2%, and specificity of 72.9%. In this algorithm, necrotizing fasciitis is suspected when there is an abnormal LRINEC score, tachycardia, hypotension, or elevated creatine kinase level.^11^ Therefore, although it has a high sensitivity, its specificity is low, making it not useful for Japanese patients.

The SIARI score had a C‐statistic of 0.769, sensitivity of 82.2%, and specificity of 67.4%. Furthermore, it includes three items that do not depend on blood tests, affected area outside the lower extremity, immunosuppressed state, and age <60 years. Although the SIARI score is useful because it allows for the suspicion of necrotizing fasciitis before blood test results are obtained, it is not a useful tool in Japanese patients as its results are comparable to those of the NEJM algorithm.^11^, ^19^

The J‐LRINEC score developed herein showed good results, with a sensitivity of 91.4%, specificity of 84.8%, and C‐statistic of 0.9683. However, the equation is complex. Therefore, in Japanese patients, the J‐LRINEC score should be used to confirm the presence of necrotizing fasciitis after LRINEC score screening. Although an imaging‐based scoring system has been proposed, magnetic resonance imaging is rarely performed in routine dermatology practice in Japan to differentiate necrotizing fasciitis from cellulitis. In fact, none of the patients in our study underwent imaging.^22^

In 2015, a modified LRINEC score was proposed that featured increased positive and negative predictive values.^18^ However, the modified LRINEC score includes pain, which is difficult to quantify, as two strong and one moderate point; therefore, we did not include it in the present study. In addition, the number of items was increased to 10, making it cumbersome and difficult to administer.

In conclusion, we validated the existing tools for differentiating cellulitis and necrotizing fasciitis in Japanese patients and confirmed the usefulness of the LRINEC score. However, the LRINEC score has low sensitivity, and its use in combination with the J‐LRINEC score developed herein may enable a more accurate differentiation between cellulitis and necrotizing fasciitis.

## CONFLICT OF INTEREST STATEMENT

None declared.

## Data Availability

The datasets generated during and/or analyzed during the current study are available from the corresponding author on reasonable request.
